# Mouse Models of Genomic Syndromes as Tools for Understanding the Basis of Complex Traits: An Example with the Smith-Magenis and the Potocki-Lupski Syndromes

**DOI:** 10.2174/138920209788488508

**Published:** 2009-06

**Authors:** P Carmona-Mora, J Molina, C.A Encina, K Walz

**Affiliations:** 1 Centro de Estudios Científicos, Valdivia, Chile; 2 Universidad Austral de Chile, Valdivia, Chile

**Keywords:** Gene copy number variation, complex traits, phenotypic consequences, mouse models.

## Abstract

Each human's genome is distinguished by extra and missing DNA that can be “benign” or powerfully impact everything from development to disease. In the case of genomic disorders DNA rearrangements, such as deletions or duplications, correlate with a clinical specific phenotype. The clinical presentations of genomic disorders were thought to result from altered gene copy number of physically linked dosage sensitive genes. Genomic disorders are frequent diseases (~1 per 1,000 births). Smith-Magenis syndrome (SMS) and Potocki-Lupski syndrome (PTLS) are genomic disorders, associated with a deletion and a duplication, of 3.7 Mb respectively, within chromosome 17 band p11.2. This region includes 23 genes. Both syndromes have complex and distinctive phenotypes including multiple congenital and neurobehavioral abnormalities. Human chromosome 17p11.2 is syntenic to the 32-34 cM region of murine chromosome 11. The number and order of the genes are highly conserved. In this review, we will exemplify how genomic disorders can be modeled in mice and the advantages that such models can give in the study of genomic disorders in particular and gene copy number variation (CNV) in general. The contributions of the SMS and PTLS animal models in several aspects ranging from more specific ones, as the definition of the clinical aspects of the human clinical spectrum, the identification of dosage sensitive genes related to the human syndromes, to the more general contributions as the definition of genetic locus impacting obesity and behavior and the elucidation of general mechanisms related to the pathogenesis of gene CNV are discussed.

## INTRODUCTION

Genomic structural changes, such as gene Copy Number Variations (CNVs) are extremely abundant among phenotypically normal human individuals [1, 2]. CNV are present in a large proportion of the human genome and include hundreds of genes [3]. Currently over 19,000 human CNVs from approximately 6,200 regions (CNVRs) have been identified, ranging from 1kb to several megabases in size (http://projects.tcag.ca/variation/). They are recognized important mutational mechanisms involved in several phenotypes that range from “normal” variability between individuals, susceptibility to traits, genome evolution, to specific human genomic disorders [4-13].

Genomic disorders are the pathological evidence of CNV and they are frequent conditions, ~1 per 1,000 births [14]. If the rearrangements are large enough to be visualized by cytogenetic techniques and comprise multiple unrelated contiguous genes they are named contiguous gene syndromes (CGS). Extensive studies have identified several CGS and despite the intra-patient variability, each CGS is defined by specific and complex phenotypes that can include neurobehavioral traits and congenital abnormalities [15-18] among other clinical presentations. The molecular mechanism prevalent for the generation of the genomic rearrangements related with CGS is nonallelic homologous recombination (NAHR) or unequal crossing over, between flanking low-copy number repeats (LCRs) [19-23], and thus the vast majority of patients with any given CGS harbor a common rearrangement encompassing a specific genomic interval.

The beauty of studying CGS is that there is a confined genomic interval that corresponds with a particular set of symptoms. Moreover, due to the mechanism by which most of the genomic rearrangements are generated it is frequent that given a specific CGS related to a deletion there will be another related to the reciprocal duplication.

Numerous new technologies were available within the last decade that allowed the assessment of CNVs within the genome, the molecular basis of mechanisms implicated in CNV generation, the discovery and study of CGS, the phenotypic outcome of a defined CGS, and the correlation between gene and phenotype. Among these new technologies the development and phenotypic assessment of mouse models for CGS have extensively contributed to this knowledge [24, 25].

## SMITH-MAGENIS (SMS) AND POTOCKI-LUPSKI (PTLS) SYNDROMES

SMS (OMIM#182290) and PTLS (OMIM#610883) represent reciprocal CGS, associated with a deletion and duplication of a 3.7 Mb genomic interval within chromosome 17 band p11.2, respectively, each having a specific clinical presentation. The SMS, has an estimated prevalence of 1/25,000 births. Whereas a commonly deleted region of ~3.7 Mb is present in the majority of SMS patients (>70-80%) [26-28], an ~1.1Mb SMS critical region was defined that includes 15 previously identified genes and eight predicted genes [29-31]. Recently, haploinsufficiency of the *Retinoic Acid Induced gene* (*RAI1*), that is located within the critical genomic interval, was associated with most features of SMS [32-35] suggesting that it is the dosage sensitive gene responsible for SMS.

Phenotypes in SMS patients are well characterized, and they are summarized in Table **1**. Clinical presentation of SMS includes obesity, craniofacial abnormalities and brachydactyly. Furthermore SMS patients show neurobehavioral phenotypes that comprise self-injurious and maladaptive behaviors, speech delay, sleep disturbances, learning difficulties and a mild to moderate range of mental retardation and seizures [36-49].

Duplication of the SMS region, resulting in the PTLS, has been reported by Potocki *et al*., initially describing seven patients [50]. Further studies redefined the clinical spectrum [51, 52] and narrowed to 1.3 Mb the critical interval responsible for the PTLS, this interval contains the *RAI1* gene [52]. The PTLS clinical phenotype includes developmental delay, mental retardation, developmental deficit for psychomotor and expressive speech, autistic features, obsessive-compulsive behaviors and attention deficit. Other clinical findings in PTLS patients encompass hypotonia, feeding difficulties in infancy, failure to thrive and EEG abnormalities (Table **1**). The dosage sensitive gene(s) responsible for PTLS remains unidentified; however, it was possible to demonstrate that CNV of the *Rai1* gene is mostly responsible for complex physical and behavioral traits in a mouse model for PTLS [53].

## DIFFERENT MOUSE MODELS FOR SMS AND PTLS

The mouse shares physiologic, anatomic and genomic similarities with humans and can be genetically manipulated [54]. It has thus become an important animal model for studying human disease. Moreover, the completion of the human and mouse genomes sequences enables comparative genomic analyses, being relatively simple to see the feasibility of the generation of a mouse model for a CGS since for this purpose aspects as the synteny of the genomic region and the order and orientation of the genes are essential information.

Depending on the original situation or question that needs to be addressed different mouse models for CGS can be developed as is summarized in Fig. (**1**). If the dosage sensitive gene is unknown but a critical genomic region is defined a genomic rearrangement (deletion or duplication) can be generated within the mouse genome. On the other hand, if the dosage sensitive gene is known a knock-out/knock-in or overexpressing transgenic mouse can be produced.

For the generation of chromosomal deletions, duplications, inversions and translocations in mice there are many methodologies described [55-58]. The common disadvantage of all these technologies is that the rearrangements are generated at random. However, we will focus in the strategy that have been developed to introduce defined chromosomal rearrangements in the mouse genome by engineering them in embryonic stem cells (ES cells) using the *Cre-loxP* site-specific recombination system [59, 60]. To generate a specific genomic rearrangement between two endpoints, two sequential gene-targeting steps are required in order to prepare each endpoint for selectable Cre-*loxP* recombination [59]. The double-targeted ES cell is then transiently transfected with Cre recombinase (an enzyme that catalyzes site specific recombination between the specific 34 bp *loxP* sites) that facilitates the recombination between the two *loxP* sites, achieving the corresponding genomic rearrangement. The type of chromosome rearrangement obtained will be a direct consequence of the relative initial *loxP* configurations. If the *loxP* sites are in the same or direct orientation, the region between them will be deleted or duplicated. If the two *loxP* sites are inverted or in opposite orientation, the genomic region between them will be inverted [61-63]. Another versatility of this methodology is the possibility to choose the desired endpoints, with the extra advantage of the availability of two complementary libraries (the 5’*Hprt* library and the 3’*Hprt* library) for the initial targeting events [60]. Actually, the Mouse Genomics – MICER project: Mutagenic Insertion and Chromosomal engineering resource (http://www.sanger.ac.uk/PostGenomics/mousegenomics/) offers this resource openly, it provide vector sequences, chromosomal location and orientation of the insertion, and information on using MICER vectors for generating knock-out mice, and for chromosome engineering purposes [64].

The above described technique was utilized to generate the SMS and PTLS mouse models. Human chromosome 17p11.2 is syntenic to the 32-34 cM region of murine chromosome 11. The number and order of the genes are highly conserved [29-31] (Fig. (**2**)). In the case of SMS and PTLS mouse models to generate the chromosomal rearrangements that include the common (syntenic) deletion region, *Csn3* was selected as the proximal anchor point for one *loxP* site and *Zfp179* was selected as the distal anchor point in murine chromosome 11. These two *loxP* sites were sequentially inserted with chromosome-engineering cassettes by gap repair recombination [60] into both anchoring points that are located approximately 3 Mb apart [30]. Double-targeted ES cells were then subjected to Cre-mediated site-specific recombination, and the ES cells carrying rearrangements were selected in HAT media by virtue of the reconstituted *Hprt* gene. In one ES cell clone both rearrangements, a deletion (*Df(11)17*) and a duplication (*Dp(11)17*) syntenic to the SMS critical region were obtained. Chimeric mice were produced and in the F1 mice heterozygous for the deletion: *Df(11)17/+* (the SMS mouse model) and mice heterozygous for the duplication: *Dp(11)17/+* (the PTLS mouse model) were segregated [65].

Another methodology that derives from the described above is the generation of nested deletions in ES cells by chromosome engineering mediated by retrovirus infection and insertion of one of the *loxP* sites in a random fashion [66]. In this case a single gene, *Csn3*, was utilized as the anchoring point by targeting it with a vector containing the *lox*P site, the 5’ *Hprt* and the neomycin resistance gene followed by an infection with retrovirus in order to introduce another *lox*P site, with the second half of *Hprt* gene and puromycin resistance gene, for selecting infected cells with this antibiotic. Again, by transfecting the selected cells with a Cre-recombinase if the specific recombination takes place the cells can be selected in HAT medium. As the integration of *lox*P sites is at random, the deletions need to be characterized by FISH, southern blot, and PCR to trace the viral DNA and the size of deletions. Three independent deletions were obtained by this methodology for the SMS region named: *Df(11)17-1, Df(11)17-2 *and* Df(11)17-3,* all of them been smaller than the *Df(11)17* [67].

In 2003, the first reports on SMS patients not harboring the common deletion in 17p11.2, but carrying mutations in the *RAI1* gene appeared [32-34]. This fact pointed to *RAI1* as the dosage sensitive gene within this genetic interval. In order to generate a mouse lacking one active allele of *Rai1* the disruption of its coding region was achieved by the in frame insertion in the exon 2, or knock–in, of the coding sequence of *E. coli lacZ* gene plus a neoR expression cassette [68]. On the other hand, a transgenic mouse carrying extra copies of a BAC containing the *Rai1* gene (RP23-326M22, GenBank accession AC096624) was generated [69]. Important is to note that this BAC clone contained not only the entire *Rai1* gene but also the complete *Srebp1* gene sequence plus the *3´*end of the *Tom1l2* gene in different number of copies that ranged from 4 to 5.

The summary of the different mouse models available for SMS and PTLS is represented in Fig. (**3**).

## LESSONS FROM THE MICE

Chronologically, the first SMS and PTLS mouse models to be generated and phenotypically assessed were the mice harboring the deletion and the duplication of the critical region, *Df(11)17/+ *and *Dp(11)17/+,* respectively [65]. The *Df(11)17/*+ mice displayed several characteristics present in SMS patients (Table **1**). Obesity was observed in these mice beginning to be markedly overweight when compared to the wild type littermates at fourth months of age; consistently, abdominal fat contents were significantly higher in these animals. Craniofacial abnormalities were also observed, presenting shorter overall skulls and broader and shorter snouts and nasal bones than the wild type mice. *Df(11)17/+* mice exhibit overt seizures (~ 20%), in concordance with the human phenotype. Moreover, an abnormal EEG pattern was detected in these mice, as well as in patients EEG records that were revised due to the mouse phenotype [45]. *Df(11)17/+* male mice were found to be hypoactive in the open field test as indicated by a significant decrease of the total distance traveled and time spent moving in the open field when compared to the wild type littermates. Sleep disturbances are a main characteristic present in the SMS patients. *Df(11)17/+* mice displayed a significantly shorter circadian period length than their wild types littermates (~27 min) when they were tested in the twenty-four hour wheel running activity test in constant darkness. More interesting was the finding that the period length distribution among *Df(11)17/+* mice was much more variable than among wild type mice, which may indicate a reduced precision of the clock control of period length associated with haploinsufficiency of genes in this defined genomic interval [70].

The mouse model for PTLS, *Dp(11)17/+* mice, also present physical characteristics of the human condition, as low corporal weight, concomitant with a significant reduction of abdominal fat content. *Dp(11)17/+ *mice presented hyperactivity as indicated by a total significant increment in the distance traveled and the time spent moving in the open field when compared to the wild type mice. Increased anxiety behavior was observed for *Dp(11)17/+* mice in the light-dark exploration test and in the plus maze test as indicated by a significant decrease in the number of light-dark transitions [70] or a time spent in the open arm [71] respectively. The conditioned fear test to assay a fear-based response using a Pavlovian learning and memory paradigm was used in *Dp(11)17/+* mice and they presented an impaired conditioned fear that was selective to the context test, and was present following both a 1 h and 24 h delay interval, indicative of a short term memory deficiency [70]. PTLS patients present autistic characteristics, in agreement with this *Dp(11)17/+* mice showed an abnormal social behavior when tested in the three chamber test for sociability or preference for social novelty [72] or the tube test (a paradigm previously found to be useful in predicting impairments in social interaction [71, 73, 74]. In summary, the *Df(11)17/+ *and *Dp(11)17/+* mice are well characterized and represent validated models for further studies (Table **1**).

When mice with smaller deletions (*Df(11)17-1, Df(11)- 17-2 *and* Df(11)17-3*) [67] were phenotypically characterized they also showed obesity and craniofacial abnormalities, but it was reported that the penetrance of the craniofacial phenotype was markedly reduced, strongly suggesting that other genes or regulatory elements influence in the penetrance of the phenotype. The genomic fragment that is different between the critical deletion (*Df(11)17*) and the shorter deletion that was analyzed in this paper was ~ 2.500 kb, comprising 14 genes, narrowing down the modifiers candidates genes.

When the *Rai^+/-^* mouse were analyzed obesity and craniofacial abnormalities were observed [68] corroborating that these two phenotypes are a direct consequence of *Rai1* gene haploinsufficiency. *Rai1*^+/-^ mutants also had an abnormal electroencephalogram with overt seizures observed in approximately 2% of the generated mice. These mice exhibited normal locomotor activity, in a mixed genetic background, in clear contrast, mice with smaller deletion (~590 kb genomic interval) and the *Df(11)17 *mice that shown hypoactivity in the open field test [75]. However, when *Rai^+/-^* mice where tested in a different genomic background the hypoactivity phenotype was recovered indicating that Rai1 is involved in these phenotype but is clearly influenced by modifiers [71].

Homozygous *Rai1^-/-^* mice displayed a more severe phenotype, presenting malformations in both craniofacial and the axial skeleton. Neurobehavioral abnormalities including hind limb clasping, overt seizures in 1/3 of the mutant mice, motor impairment, and context and tone dependant learning deficits were also present in these mice [68, 75]. Another advantage of this mouse model was that the reporter *lacZ* gene was knocked-in the *Rai1* locus making it possible to study the expression patterns of *Rai1* during the embryogenesis and adulthood. When the blue staining, resulting from the β-galactosidase activity, indicative of *Rai1* gene expression was studied during embryogenesis it was found that at 9.5 dpc *Rai1* is expressed mainly in branchial arches and otic vesicles. Postnatal studies showed low levels of expression throughout the brain, being most predominantly expressed in the hippocampus and the cerebellum. In addition to the brain, *Rai1* expression was found in thymus, bronchioles of the lung, collecting tubules in the kidney, the testis, the epididymis, and the ovaries, the hair follicles in the skin, the tongue, the mandibular gland and the intestine [75]. Another important finding obtained from this model is that *Rai1* has an important participation in embryonic development, since *Rai1*^-/-^ mice presented impaired viability, and growth retardation of the survived mice [68]. Moreover, the *Rai^+/-^* survival rate was found to be dependent of genetic background [76] something that was also observed for the *Df(11)17/+* mice [77].

In a first attempt to identify the gene responsible for the PTLS a study was carried out with *Dp(11)17/Rai1^-^* compound heterozygous mice, since both mouse models were already available. By mating *Dp(11)17/+* mice with *Rai1^+/-^* animals we were able to analyze *Dp(11)17/+*, wild type, *Rai1^+/-^* and *Dp(11)17/Rai1^-^* mice and explore the role of *Rai1* copy number in the *Dp(11)17/+* phenotype [53]. The generation of *Dp(11)17/Rai1^-^* compound heterozygous animals, determined that restoring the 2n gene dosage of *Rai1* alone is sufficient to rescue part of the phenotypes observed in the PTLS mouse model (weight, anxiety and learning difficulties), despite the trisomic copy number of the remaining 18 genes in the SMS critical interval (Table **2**) (Fig. (**4**)). Thus, it was demonstrated that *Rai1* was the gene whose CNV within this genomic interval was responsible for several complex physical and behavioral traits. However, is important to point out here that not all the phenotypes observed in the *Dp(11)17/+* mice were rescued by the correction of *Rai1* gene dosage (see below).

Years later, and reinforcing the concept that *Rai1* was the dosage sensitive gene responsible for PTLS, transgenic mice containing four or five additional copies of *Rai1* were generated and tested for assessing physical, neurological and behavioral phenotypes. These transgenic mice were significantly underweight and got smaller head and body sizes when compared with their wild type littermates. Hyperactivity, increased anxiety in the open field and a dominant behavior in the tube test for social interaction evaluation were also observed in the transgenic mice. Abnormal maternal behavior, altered sociability, and impaired serotonin metabolism in *Rai1*- BAC transgenic mice with a dosage-dependent worsening of the phenotype while increasing copies of *Rai1,* was also reported [69, 78].

As was previously described most but not all phenotypes were rescued in the *Dp(11)17/Rai1^-^* mice. This prompted to the evaluation of *Dp(11)17/Df(11)17* phenotype. Since the same genomic region is deleted and duplicated, these mice represented a unique tool in order to study the consequences of restoring the correct gene copy number within the interval, albeit in a uniallelic fashion. All these experiments were performed in an inbred genetic background. Essentially the same battery of experiments for assessing the phenotypes before mentioned, were tested in mice with 1n (*Df(11)17/+)*, 2n (wild type and *Dp(11)17/Df(11)17)*, or 3n (*Dp(11)17/+)* copies of the genes within the critical genomic interval. It was found that craniofacial abnormalities observed for *Df(11)17/+* were completely rescued in *Dp(11)17/Df(11)17* mice, demonstrating that this phenotype is due to the haploinsufficiency of the genes deleted within the SMS region. This dosage restoration also rescued the differences of body weight between *Df(11)17/+* and *Dp(11)17/+* mice, since the animals *Dp(11)17/Df(11)17* have wild type weight curves. The behavioral traits of *Dp(11)17/Df(11)17* progeny, in which the dosage of all 19 genes in the SMS critical interval is balanced, were mostly normalized (seizures, anxiety, learning and memory) (Table **2**), indicating that gene dosage, more than positional effects, influences behavior. This is true for most of the phenotypes, however, the hyperactivity shown in the open field by *Dp(11)17/+* mice was not rescued in *Dp(11)17/Df(11)17* mice [53]. In conclusion, phenotypic assessment of *Dp(11)17/Df(11)17* animals, in a pure genetic background, demonstrate that some phenotypes observed in CGS are due by “dosage effect” but others are a consequence of “positional effect”, uncoupling, for the first time, the effects of gene CNV and genomic structural changes and highlight the delicacy of genomic control mechanisms [53, 77].

Another interesting aspect that can be addressed by studying these mouse models is the influence of structural changes on the transcription of genes within and outside the rearranged interval. It was previously reported that the aneuploid genes and also the flanking genes that map up to several megabases away from the rearranged interval are affected in their relative expression level for the human chromosome 7 DNA deletion that causes Williams-Beuren syndrome [79]. By the study of the hippocampus transcriptome of *Dp(11)17/+* male mice compared to their normal littermates by real time PCR and DNA microarrays, [71] it was found that not only the genes included in the genomic rearranged region, but also normal copy number genes that flanked the engineered interval showed altered (up or down) expression levels in the hippocampus of *Dp(11)17/+* mice. Analysis of the transcriptome of *Df(11)17/Dp(11)17* mice is underway, but preliminary data shows that these “flanking” effects are unidirectional and uncoupled from the number of copies of the CNV genes (personal communication). Thus, these results indicate that a structural change at a given position of the genome may cause the same perturbation in particular pathways regardless of gene dosage. Moreover, results reported in wild mice and classical inbred strains where a CNV map was generated and the genome-wide expression data from six major organs was compared showed that not only the expression of genes within CNVs tend to correlate with copy number changes, but also that CNVs influence the expression of genes in their vicinity, an effect that extends up to half a megabase [80, 81]. Taken these results altogether suggest that this kind of genomic variation plays a general role in the phenotypic outcome.

In summary, although extensive research on CNV is taken place in a genomic context, mouse models have prove to be an efficient and unique tool to unravel the phenotypic and molecular consequences of genomic disorders. These advantages includes more specific aspects related to the syndromes to which they are associated as the discovery of dosage sensitive genes, the definition of the phenotypic consequences of the rearrangement, the study of the function of genes affected by CNV and their participation in a specific phenotype to more general aspect as the contribution in the understanding of the effects of CNV in a genomic context.

## Figures and Tables

**Fig. (1).**
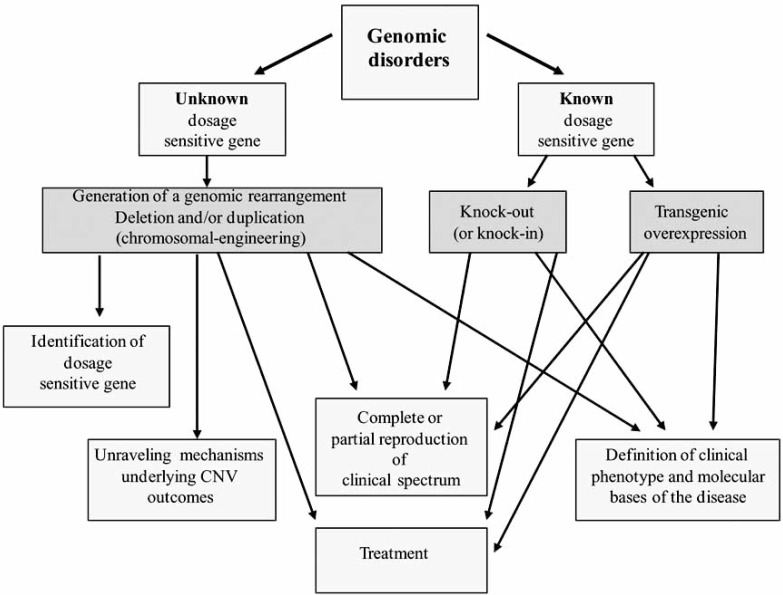
**Steps to consider when modeling genomic disorders in mice.** A graphic representation of the steps to follow when deciding to generate a mouse model for CGS is presented. Different strategies could be followed depending if the dosage sensitive gene is known or unknown. Multiple advantages can be obtained with the different models as listed in the figure.

**Fig. (2).**
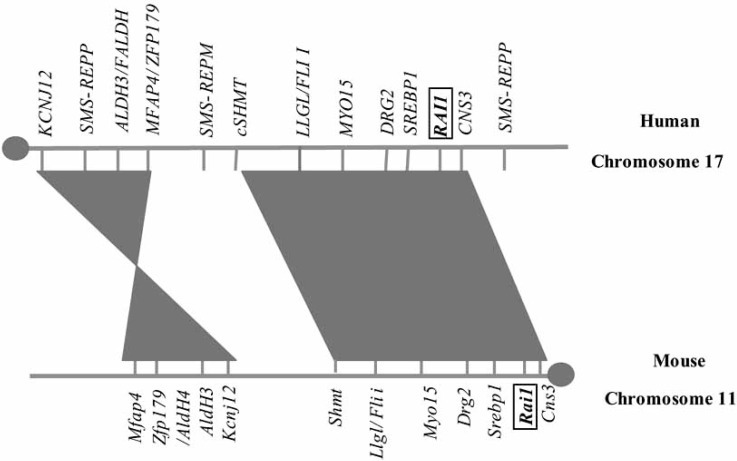
**Schematic representation of the SMS and PTLS genomic region.** Representation of the 17p11.2 region of human chromosome 17 and its syntenic counterpart region 32- to 34-cM of the mouse chromosome 11 is depicted [53]. Centromeres are represented in gray circles. Note that the numbers, orientations, and relative orders of the genes in these syntenic genomic intervals are extremely conserved. Three low-copy repeats are present in the human region: SMS-REPD (distal); SMS-REPM (middle); SMS-REPP (proximal).

**Fig. (3).**
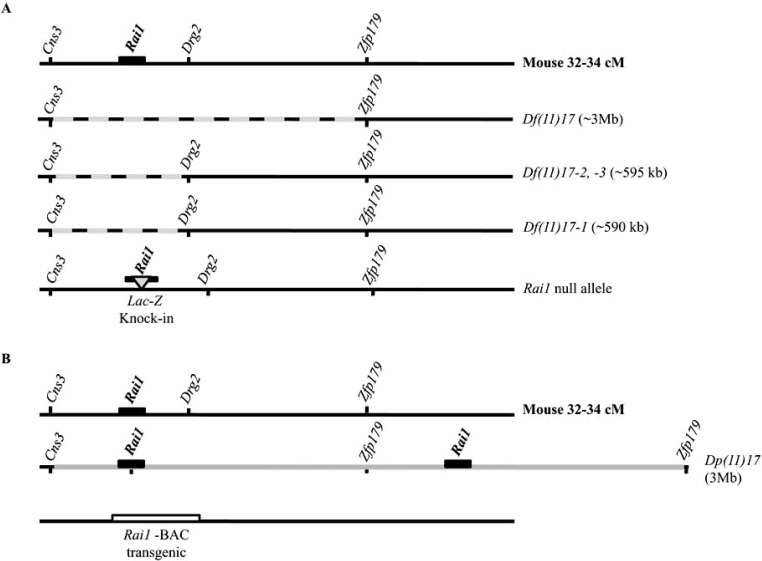
**Existing mouse models of SMS and PTLS syndromes. A**) Schematic representation of the wild type 32-34 cM region of 11 mouse chromosome (black line, Mouse 32-34 cM) and the different deletions available at the present (black-grey doted line). The SMS critical region (*Df(11)17*) [65] and the smaller deletions (*Df(11)17-1, -2, -3*) [67] are depicted. Finally the mouse harboring a *LacZ* knock-in in the *Rai1* coding sequence is illustrated with a light grey triangle (*LacZ* knock-in) [68]. **B**) Representation of the two murine models for the PTLS, one carrying the duplication of the SMS critical region in 11 chromosome 32-34 cM (dark grey line)[65] and a BAC transgenic mouse containing the *Rai1* gene [69]. The sizes of the rearrangements are specified within parenthesis.

**Fig. (4).**
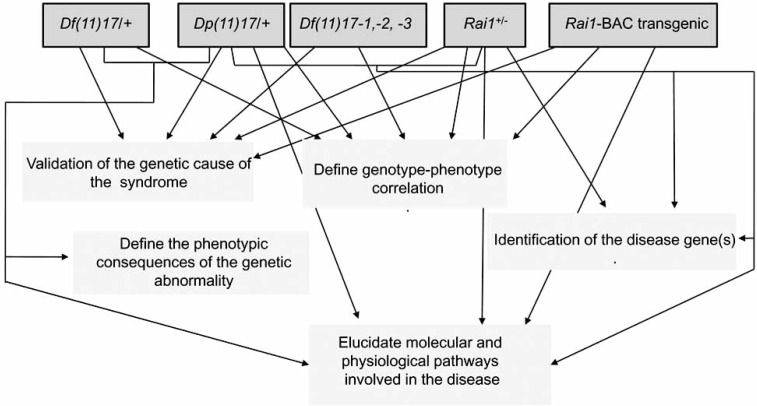
**Contribution of existing mouse models for SMS and PTLS.** The contribution of the study of the existing SMS and PTLS mouse models in the understanding of the phenotypic and molecular consequences of genomic disorders.

**Table 1.** Phenotypes Present in SMS and PTLS Patients and in their Respective Mouse Models

Phenotype	SMS	*Df(11)17/+*	PTLS	*Dp(11)17/+*
**Body Weight**	Elevated	Overweight	Reduced	Underweight
**Craniofacial/Skeletal**	Abnormal	Abnormal	Abnormal	Normal
**Speech**	Delay, “Horse voice”	ND	Altered	ND
**Overt Seizures**	Present (~20%)	Present (~20%)	Absent	Absent
**EEG**	Abnormal	Abnormal	Abnormal	Normal
**Locomotor Activity Levels**	Motor delay	Hypoactive	Hyperactive	Hyperactive
**Main Behavior Abnormality**	Self injurious	Normal	Autistic	Social abnormalities
**Learning and Memory**	Mental retardation	Normal learning and memory	Mental retardation	Abnormal learning and memory
**Pain Sensitivity**	Decrease	Normal	Normal	Normal
**Sleep**	Abnormal	Abnormal circadian period	Normal	Normal

**Table 2.** Phenotypes Present in Mice with the Complete Gene Dosage Corrected within this Genomic Interval (*Dp(11)17/Df(11)17*) or Only *Rai1* Gene Dosage Corrected (*Dp(11)17/Rai1^-^*) Compared to *Dp(11)17*/+ and *Df(11)17*/+ Mice

Phenotype	*Dp(11)17/+*	*Df(11)17/+*	*Dp(11)17/Df(11)17*	*Rai1^+/-^*	*Dp(11)17/Rai1^-^*
**Body weight**	Reduced	Higher	Normal	Higher	Normal
**Overt seizures**	Absent	Present (20%)	Absent	Subtle (2%)	Absent
**EEG**	Normal	Abnormal	Abnormal	Abnormal	ND
**Locomotor activity**	Hyperactive	Hypoactive	Hyperactive	Normal/Hypoactive(***)	Hyperactive
**Anxiety**	Increased*	Decreased*	Decreased*	Normal**	Normal**
**Learning and memory**	Impaired	Normal	Normal	Normal	Normal
**Citation**	[65, 70]	[65, 70]	[53, 65]	[53, 68, 75]	[53]

(*) Phenotype was tested by plus maze test (data non published).

(**) open field.

(***) Depending on the genetic background.

## ABBREVIATIONS

BAC: = Bacterial Artificial Chromosome
CGS: = Contiguous Gene Syndromes
CNV: = Copy Number Variation
FISH: = Fluorescence *in situ* Hybridization
HAT: = Hypoxanthine, Aminopterin, and Thymidine medium
*Hprt*: = Hypoxanthine-guanine phosphoribosyltransferase gene
LCRs: = Low-Copy Number Repeats
MICER: = Mutagenic Insertion and Chromosomal Engineering Resource
NAHR: = Nonallelic Homologous Recombination
ND: = Non Determined
PTLS: = Potocki-Lupski Syndrome
*RAI1*: = Retinoic Acid Induced 1
SMS: = Smith-Magenis Syndrome
SMS-REPD: = Smith-Magenis Syndrome- low copy repeat distal
SMS-REPM: = Smith-Magenis Syndrome- low copy repeat middle
SMS-REPP: = Smith-Magenis Syndrome- low copy repeat proximal
